# Relearning the epistemology, history, and future of neuropsychiatry

**DOI:** 10.3389/fnhum.2026.1727506

**Published:** 2026-03-02

**Authors:** Juan Camilo Castro Martínez, Felipe Botero-Rodríguez, Jesús Ramírez-Bermúdez, Vaughan Bell, Gabriel Oviedo-Lugo, José Manuel Santacruz-Escudero, Ángela Iragorri, Joan Camprodon, Brian Lawlor, Hernando Santamaría-García

**Affiliations:** 1 Departamento de Psiquiatría y Salud Mental, Facultad de medicina, Pontificia Universidad Javeriana, Bogotá, Colombia; 2 Center for Memory and Cognition, Intellectus, Hospital Universitario San Ignacio, Bogotá, Colombia; 3 SynaptIA- Inteligencia Artificial para la investigación en salud mental, Bogotá, Colombia; 4 Instituto de Envejecimiento, Facultad de medicina, Pontificia Universidad Javeriana, Bogotá, Colombia; 5 National Institute of Neurology and Neurosurgery, Universidad Autonoma de México, Mexico City, Mexico; 6 University College of London, London, United Kingdom; 7 Division of Neuropsychiatry & Neuromodulation, Massachusetts General Hospital, Harvard Medical School, Boston, MA, United States; 8 Trinity College, Dublin, Ireland; 9 Global Brain Health Institute (GBHI), University California San Francisco, San Francisco, CA, United States

**Keywords:** deep phenotyping, dimensional psychiatry, mind–body integration, neurophenomenology, neuropsychiatry

## Abstract

Neuropsychiatry is a transdisciplinary field at the intersection of neuroscience, psychiatry, neurology, and humanities. Despite this strategic position, a comprehensive framework is still needed to bridge these domains. This review examines the historical evolution of how neurological, mental, and neuropsychiatric symptoms have been conceptualized, from antiquity to contemporary models, using the brain–body dilemma as a guiding thread. This historical analysis provides the epistemological and ontological foundations of neuropsychiatry, which are then connected with current definitions to critically assess the field's persistent tensions. Building on this foundation, a renewed paradigm is proposed where a crosstalk between them is enabled, grounded in deep phenotyping, dimensional research frameworks [e.g., Research Domain Criteria (RDoC), Hierarchical Taxonomy of Psychopathology (HiTOP)], and integrative models linking biological, psychometric, social data, and subjective experience. Special attention is given to a “subjectomic” layer that aims to systematically incorporate lived experience. Finally, reforms in education, clinical practice, and research are advocated to foster this conceptual reorientation, aiming at interdisciplinary collaboration and advancing patient care.

## Introduction

Neuropsychiatry is a discipline at the crossroads of medicine, neurology, psychiatry, neuroscience, psychology, physiology, phenomenology, and philosophy. It investigates behavioral abnormalities, cognitive dysfunction, and mental symptoms in neurological and somatic conditions, while also advancing neurobiological explanations of psychiatric disorders (Berríos, 2007). Yet, defining its scope remains difficult (Sachdev P. S., 2005; Berríos, 2007; Poole and Bolton, 2020), and it is often described as a “no man's land” between neurology and psychiatry (Sachdev P. S., 2005).

Neuropsychiatric disorders are highly prevalent and rank among the leading global causes of disease burden (Nichols et al., 2019; Ferrari et al., 2022; Steinmetz et al., 2024). Conditions such as stroke, migraine, dementias, epilepsy, and autism account for significant disability-adjusted life-years (DALYs; Steinmetz et al., 2024), and depressive and anxiety disorders, including somatic equivalents like pain and insomnia, rank among the top contributors to DALYs worldwide (Ferrari et al., 2022). Their impact extends beyond tissue damage, affecting cognitive, affective, behavioral, social, and functional domains.

Despite progress, neuropsychiatry faces persistent theoretical and pragmatic challenges, including a lack of consensus on the study and care of affected patients, and the absence of a unified praxis or epistemological framework (Ramirez-Bermudez et al., 2017). In part, this is based on the epistemological difficulties encountered in psychiatry, where the object of study is defined by complex interplay between social, political, historical processes and folk psychology dynamics (Berrios and Marková, 2018). Parting from that point, neuropsychiatry puts forward a critical point, the need for dialogical bridges to give responses to clinical and research questions. These gaps have led to fragmented clinical and research approaches, shaped by enduring tensions surrounding the body–mind problem (Thibaut, 2018).

This paper offers an overview of neuropsychiatry: the historical evolution of symptom conceptualization describing the tensions between mental and neurologic symptoms both at epistemological and ontological levels; its epistemological bases, current definitions, and future directions. Although these developments are presented sequentially to support the subsequent epistemological discussion, such structure does not imply a linear or cumulative progression of knowledge, which often unfolds through non-continuous and contingent processes (De Domenico et al., 2016). This review highlights shifts in neurological and mental concepts, the persistence of the mind–body dilemma, and the role of philosophy and technology in shaping research and care. Finally, we discuss challenges in clinical, academic, and training contexts, proposing an integrative orientation aimed at translating neuroscientific knowledge and subjective understanding into innovative practice.

### Historical background of mental and neurological symptoms

It is important to acknowledge at the outset that the historical literature most relevant to neuropsychiatry is underdeveloped, although some general aspects of its historical development can be discerned (Scull, 2017). In ancient Greece, neurologic and psychiatric symptoms were viewed as a single domain. Guided by the Hippocratic humoral theory, behavior and mental states were interpreted as manifestations of bodily imbalance (Trimble, 2016). In the 16th century, Andreas Vesalius and Thomas Willis re-anchored mind and behavior in brain biology, although humoral and ventricular doctrines persisted (Trimble, 2016). The 17th century brought systematic case descriptions by Thomas Sydenham and René Descartes' substance dualism, which formalized the mind–body split still echoing in today's debates (Trimble, 2016; Thibaut, 2018). Willis emphasized brain matter over ventricles and proposed “animal spirits” as mediators of mental life (Trimble, 2016). John Locke reframed mental symptoms as cognitive phenomena (Trimble, 2016), and Karl Jaspers later established phenomenological psychopathology, stressing first-person experience (Park, 2019).

By the late 19th century, figures such as Emil Kraepelin and Jean-Martin Charcot contributed to the institutional consolidation of psychiatry and neurology as distinct disciplines; however, this divide cannot be attributed solely to their work: Kraepelin through classification systems grounded in psychopathology (Heckers et al., 2022), and Charcot through the anatomo-clinical method, correlating clinical signs with post-mortem findings (Drouin et al., 2022). Yet, the divide was not absolute; neurology remained highly influential for the development of modern psychiatry from alienism (Bogousslavsky and Moulin, 2009), and scholars like Hughlings Jackson proposed hierarchical models of brain function, emphasizing integration rather than localization (Martin, 2002; Trimble, 2016). Furthermore, there were notable alienists who contributed to the study of the nervous system, effectively serving as early “neuropsychiatrists,” such as Baillarger and Lasègue (Bogousslavsky and Moulin, 2009).

The early 20th century saw major advances in neurobiology by Santiago Ramón y Cajal, Franz Nissl, and Alois Alzheimer, alongside a growing specialization in medicine, particularly in the US (Goetz et al., 2003). Clinicians increasingly distinguished between cases with clear neuropathology and those with primarily behavioral symptoms (Tyler et al., 2003).

French scholars countered this emerging binary. Paul Lhermitte and Julian de Ajuriaguerra promoted body–mind unity, while Henri Ey proposed a hierarchical model linking localized (neurological) and global (psychiatric) disintegration (Drouin et al., 2022). Nonetheless, France abolished neuropsychiatry in 1968 amid psychodynamic and sociopolitical pressures (Drouin et al., 2022). In contrast, Alexander Luria advanced a synthesized neuropsychological model (Peña-Casanova et al., 2024), and Germany retained combined training (Drouin et al., 2022).

Global conflicts, particularly World War I and II, catalyzed the recognition of combat-related mental and neurological conditions, advancing their conceptualization and care. During World War I, the US Army created neuropsychiatric units at the front lines, marking the first formal use of the term neuropsychiatry in 1917 (Crocq and Crocq, 2000; AMEDD Center of History and Heritage, n.d.). These developments laid the foundation for specialized battlefield care and institutional frameworks. In 1933, the American Board of Psychiatry and Neurology (ABPN) was founded to certify training in neurology, psychiatry or both in the US (Benjamin, 2024). After World War II, however, the trend shifted toward specialization, as the World Health Organization (WHO) and the World Psychiatric Association (WPA) endorsed separating neurology and psychiatry into distinct disciplines in 1963 and 1966, respectively (Estingoy, 2019).

The first edition of the Diagnostic and Statistical Manual of Mental Disorders (DSM) in 1952 categorized psychiatric conditions as either organic or non-organic (Fischer, 2012). Psychiatry's alliance with psychoanalysis widened the gap (Berrios and Marková, 2002). This is also reflected in the decreased of dual-boarded neuropsychiatrists observed after the creation of the ABPN, especially in the 60's and 70's (Benjamin, 2024).

The 1980s reopened integration. DSM-III adopted an atheoretical, symptom-based approach (Wilson, 1993; Fischer, 2012). The American Neuropsychiatric Association (ANPA, 1988) and UK neuropsychiatric centers, staffed by dual-trained physicians, created a clinical “third space” (Coffey, 1999). In parallel, the British Neuropsychiatry Association (BNPA, 1987) was founded to promote interdisciplinary dialogue among psychiatrists, neurologists, neuropsychologists, and other neuroscience professionals interested in brain–behavior relationships (Agrawal et al., 2015; Bhattacharya et al., 2015).

Parallel advances in cognitive science and neuroimaging reframed many psychiatric disorders as brain-based (Bolton, 2013; Tian et al., 2023). Yet, as Eric Kandel noted, articulating a model intelligible to both neuroscientists and psychiatrists remains the core challenge (Kandel, 1998). Despite this progress, the development of neurodiagnostic tools and academic structures continues to reinforce separation, perpetuating a dualistic paradigm that still hinders interdisciplinary integration (Tyler et al., 2003; Thibaut, 2018).

Since the early 2000s, technology and large-scale research initiatives have reshaped the neuropsychiatric landscape, helping bridge neurology and psychiatry. Functional MRI (fMRI), PET, SPECT, and high-resolution MRI have mapped brain activity with unprecedented precision (Tu et al., 2021; Tozzi et al., 2024). These tools have revealed overlapping neural circuits implicated in both psychiatric and neurological conditions (Tu et al., 2021).

The Human Connectome Project and other large-scale neuroimaging studies have standardized multimodal brain mapping, showing how traditionally “psychiatric” and “neurological” disorders affect common neural pathways (Thompson et al., 2020; Tu et al., 2021). Computational psychiatry has begun to integrate multi-level perspectives of neuropsychiatric disorders (Castro Martínez and Santamaría-García, 2023).

Genetic imaging consortia, such as ENIGMA and PsychENCODE, have identified polygenic overlaps between epilepsy, schizophrenia, and bipolar disorder (Thompson et al., 2020), reinforcing the convergence of psychiatric and neurological disease architectures. Conceptual models such as the Research Domain Criteria (RDoC) and Hierarchical Taxonomy of Psychopathology (HiTOP) frameworks further encourage a dimensional, brain-behavior-based understanding of mental illness (Michelini et al., 2021). Digital phenotyping through smartphones and wearable technologies now offers real-time behavioral markers that can inform diagnosis and relapse prediction in both mood and seizure disorders (Onnela and Rauch, 2016).

Contemporary neuropsychiatry increasingly integrates connectomic models with active inference frameworks (Friston et al., 2017) and constructivist approaches to behavior and mind (Barrett, 2017) reflecting a paradigm shift toward multiscale, embodied perspectives. These models converge on the idea that mental functions emerge from complex, dynamic patterns of brain connectivity rather than isolated regions. At the same time, they emphasize that cognition, emotion, and behavior are constructed from the interplay between neural architecture, bodily biology (e.g., inflammation, autonomic, and metabolic systems), and contextual interactions with the world. This integrated perspective moves beyond reductionist, dualistic models, highlighting how mind and psychopathology arise from distributed, brain–body–environment systems (Santamaría-García et al., 2024).

Rather than offering a chronological account of neuropsychiatry as a discipline, the preceding section traced how mental and neurological symptoms have been framed across time, highlighting the cultural and scientific influences that shaped their meaning and exposed enduring conceptual tensions. Some caveats must be mentioned as this description is linear since it is not the aim of the review to give a thorough historical recount but to give a succinct recount of how the object of study of neuropsychiatry has been shaped by historical processes involving cultural factors and philosophical currents. This historical lens provides the basis for reexamining the epistemological foundations and ontological concepts of neuropsychiatry, enabling a better understanding of neuropsychiatry as previously Berríos and Markova have stated that this understanding requires an understanding of the history and the idiographic needs (Berrios and Marková, 2024).

### Unraveling definitions, global variations, and debates in neuropsychiatry

**Figure 1:**
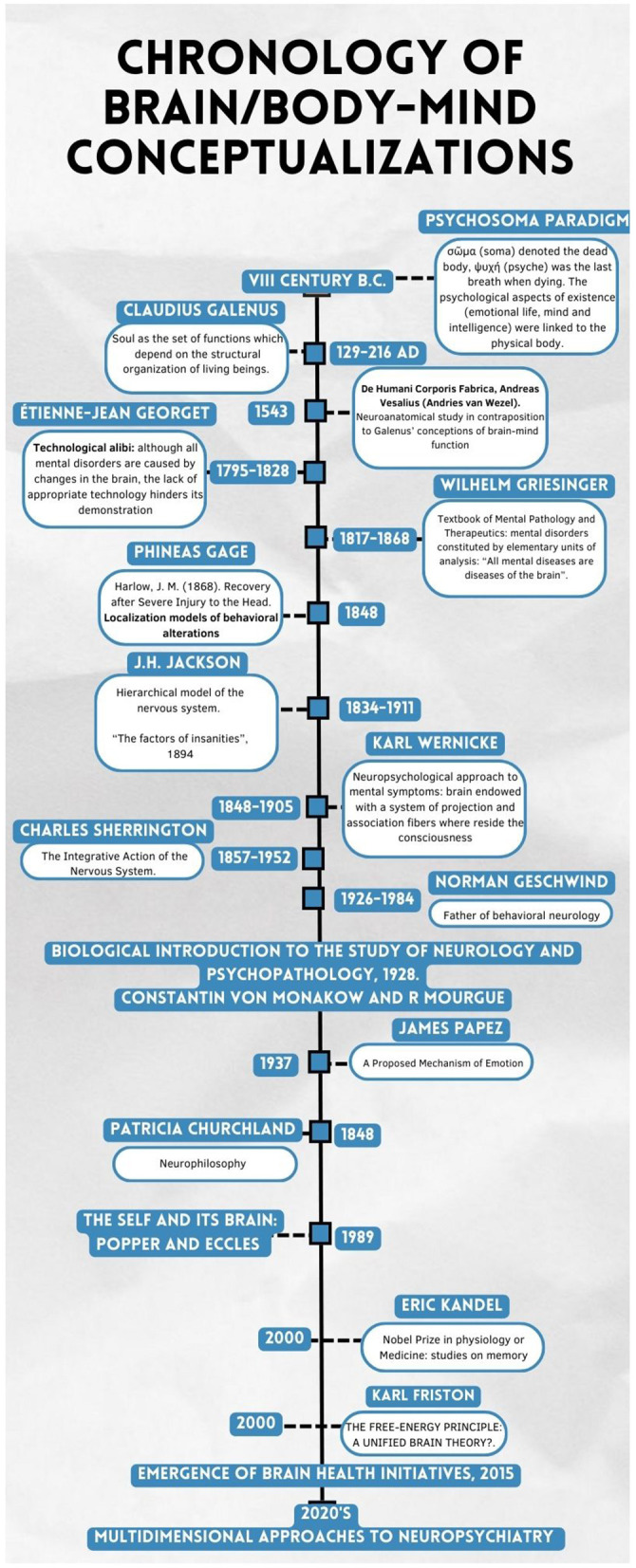
Chronology of brain/body-mind conceptualizations as a means to understand neuropsychiatry. Key milestones in the framing of neurological and psychiatric symptoms. This historical overview highlights enduring tensions in the brain–body–mind relationship that continue to shape neuropsychiatry.

The evolving conceptions of the brain–mind relationship continue to shape epistemological practices in neurology, psychiatry, and neuropsychiatry by mediating at the same time the ontology (Figure 1), influencing how disciplinary boundaries are drawn. Philosophical divergences historically contributed to separating neurological from mental symptoms, whereas moments of convergence fostered a “third space” connecting them. Building on these ideas, this section reviews contemporary definitions of neuropsychiatry to clarify its epistemological scope and present-day challenges (Poole and Bolton, 2020).

The International Neuropsychiatric Association defines neuropsychiatry as “a field of scientific medicine that concerns itself with the complex relationship between human behavior and brain function, and endeavors to understand abnormal behavior and behavioral disorders based on an interaction of neurobiological and psychological–social factors” (Sachdev P., 2005). This positions neuropsychiatry as a broad scientific domain, rather than a purely clinical specialty, focused on behavior–brain interactions and mind–body interfaces.

ANPA, in turn, describes neuropsychiatry as both a scientific field and clinical subspecialty targeting mental disorders linked to nervous system disease (Sachdev and Mohan, 2017). The Joint Committee on Subspecialty Certification of the ANPA and the Society for Behavioral and Cognitive Neurology (SBCN) have explicitly stated the core philosophical position that brain and behavior are inseparable by merging historically separate but parallel disciplines of behavioral neurology and neuropsychiatry into one subspecialty (Arciniegas and Kaufer, 2006). This medical subspecialty aims to improve understanding of the links between neuroscience and behavior and to advance the care of individuals with neurologically based behavioral impairments (Arciniegas and Kaufer, 2006).

Globally, neuropsychiatry's structure varies, however, there is an agreement of insufficient integration at training (Molina-Ruiz et al., 2024). In the US, dual training programs in psychiatry and neurology and formal fellowships have made it a leading center for neuropsychiatric research and practice (Arciniegas and Kaufer, 2006; Benjamin, 2024). In Australia and New Zealand, neuropsychiatry services are based in tertiary hospitals and focus on epilepsy, neurodevelopmental disorders, traumatic brain injury, and Huntington's disease. Care often includes multidisciplinary approaches with neurostimulation and rehabilitation, and telehealth is used to reach remote areas (Finucane et al., 2020).

In Europe, the field is more heterogeneous. In France, Henry Ey's organo-dynamic model (Drouin et al., 2022) and sociopolitical movements led to the dissolution of neuropsychiatry and the establishment of separate neurology and psychiatry tracks (Estingoy, 2019; Drouin et al., 2022). Germany, in contrast, retained a unified discipline (“Nervenheilkunde”) and strong ties to psychosomatic medicine (Northoff, 2008). The UK maintained neuropsychiatry services rooted in the work of Hughlings Jackson and W. A. Lishman, including master's-level training and extensive research on care models (Agrawal et al., 2015; Bhattacharya et al., 2015).

In Japan, the aging population has driven development in neuropsychiatry with significant overlap with geriatric psychiatry (Miyoshi, 2020). Academic neuroscience in Japan has linked neurological and psychiatric research, with contributions on the behavioral effects of environmental exposures like heavy metals.

In many Low- and Middle-Income Countries (LMICs), neuropsychiatry lacks formal institutional support. In Southeast Asia, training is limited to high-complexity centers and often consists of cross-specialty placements between neurology and psychiatry (Krishnamoorthy and Misra, 2020). Despite this, recovery-oriented programs emphasizing cultural and functional adaptation and psychosocial support have emerged (Krishnamoorthy and Misra, 2020). In Latin America, Argentina and Mexico have had neuropsychiatry advances as collaborations among behavioral neurology institutes and academic centers (Ramirez-Bermudez et al., 2017; Slud Brofman and Brusco, 2020). In general, in LMICs, neuropsychiatric expertise has developed within consultation–liaison and clinical neuroscience models, often focused on dementia diagnostics, functional neurologic disorder clinics, epilepsy and movement disorders programs (Krishnamoorthy and Misra, 2020; Slud Brofman and Brusco, 2020).

Beyond clinical and organizational variations, fundamental ontological debates remain central to neuropsychiatry. These debates, rooted in longstanding questions about the mind–body relationship, shape how neuropsychiatry defines its scope, methods, and prospects for integration. Mind–body substance dualism, the philosophical view that mental phenomena are ontologically distinct from physical processes, has profoundly shaped psychiatry and neurology by historically encouraging the separation of mental disorders from neurological disease. The nature of the relationship between these ontological domains remains contested (Berrios, 2018; Thibaut, 2018).

Materialism posits that mental phenomena must ultimately be explained or reduced to physical processes (Poole and Bolton, 2020), identifying neurobiological substrate as the necessary level of explanation for apparently mental phenomena (Van Oudenhove and Cuypers, 2010). Eliminative materialism additionally argues that mental constructs are incoherent folk concepts that will eventually be eliminated with neuroscience as the sole viable level-of-explanation for experience and behavior (Churchland, 1981). These reductionist and eliminativist approaches are hard to reconcile with the need to deal with lived experience as it presents clinically and so their applicability to psychiatry remains limited. Interactionist approaches (Berrios, 2018), recognizing both brain-mind and mind-brain causality, and patchy reductionism (Kendler, 2005), where mental phenomena can be partially reduced to biological mechanisms in some domains, while in others, requiring irreducibly psychological or social explanations, are more widely cited as a fruitful philosophical basis for neuropsychiatry.

A substantial portion of ontological reflection concerns the nature of the mind itself. Functionalism conceptualizes mental states as functional systems defined by the relations among mental functions. While grounded in physical structures, the emphasis lies on the organization and interdependence of functional relations (Levin, 2023), highlighting their relational character (Van Oudenhove and Cuypers, 2010) and dependence on physical substrates. Epiphenomenalism also acknowledges such dependence but conceives mental symptoms as secondary by-products of brain activity, lacking causal influence (Van Oudenhove and Cuypers, 2010). In contrast, emergentism grants mental states an autonomous causal powers, beyond mere dependence, allowing them to influence physical domains (Kim, 1999; Van Oudenhove and Cuypers, 2010). Other positions, such as Hughlings Jackson's parallelist perspective, deny any interaction between mental and physical domains (Berrios, 2018), challenging the possibility of integrating neurology and psychiatry (Pies, 2005).

Beyond these ontological debates, neuropsychiatry's epistemological foundations draw on diverse philosophical and scientific traditions. Naturalized epistemologies view neuropsychiatric knowledge as grounded in empirical and neuroscientific investigation, assuming that understanding of mental phenomena arises from biological observation and experimentation (Northoff, 2022; Ramírez-Bermúdez et al., 2024). In contrast, constructivist and critical epistemologies highlight that concepts such as *mental disorder* or *brain dysfunction* are historically and socially mediated, shaped by prevailing paradigms rather than discovered as fixed entities (Slade, 2012). Hermeneutic and phenomenological traditions further emphasize the experiential and interpretive dimensions of clinical understanding (Parnas et al., 2013; Aragona and Marková, 2015). Pluralistic and non-reductive epistemologies argue that metaphysical, phenomenological, and ethical dimensions cannot be disentangled from the study of brain–behavior relationships (Northoff, 2014).

The interplay between ontological and epistemological dimensions is especially evident in the study of mental symptoms within neuropsychiatry. Addressing this interdependence requires an integrative framework capable of accommodating complexity, dynamism, and multilevel and transdisciplinary explanations in both neurological diseases and mental disorders. Several challenges must be met to ensure translational validity: the problem of contested concepts, which requires the neurobiopsychosocial model to adopt a truly systemic perspective that bridges the diverse cultures of psychiatry; the inherent complexity of mental symptoms; and the value-ladenness embedded in their interpretation (Fulford et al., 2014). Consequently, it is essential to acknowledge the continuum between empirical sciences and philosophy, fostering an enriched dialogue that supports the development of renewed conceptualizations (Klar, 2021).

### Contemporary neuropsychiatry, challenges and pitfalls

While international and national associations have contributed to defining and expanding neuropsychiatry, refining epistemological and clinical approaches across neuroscience, psychiatry, and neurology (Berrios and Marková, 2002; Bhattacharya et al., 2015), key limitations persist. Considering neuropsychiatry, and psychiatry more broadly, solely as a branch of medicine constrains the potential for a contextualized understanding of mental symptoms, which extends beyond statistical correlations of proxy variables (Berrios and Marková, 2024).

Despite a growing conceptual overlap between mind and brain functions, integration between neurology and psychiatry remains incomplete (Martin, 2002; Pies, 2005). This reflects limitations in current conceptual heuristics, which inadequately capture the complexity of mental symptoms, particularly their pleiotropic and heterogeneous nature (McGorry et al., 2025). Furthermore, the persistent difficulty in achieving a shared ontological understanding of mental symptoms across disciplines generates parallel epistemological approaches, producing disparate narratives that hinder deeper integration and compromise translational validity (Fulford et al., 2014). These issues underscore the need for a constructivist epistemic framework that can offer coherence without presupposing a single reductive ontology.

Clinical practice continues to reflect this dualism, although training environments have shown increased convergence, albeit without a distinct neuropsychiatric curriculum (Molina-Ruiz et al., 2024). Neurological and psychiatric conditions share a substantial disease burden (Nichols et al., 2019; Ferrari et al., 2022; Steinmetz et al., 2024), and their comorbidities are poorly quantified and insufficiently addressed, impairing efforts to provide unified care and accurately assess the full impact on quality of life and disease progression (Taslim et al., 2024).

Neurology relies on structured diagnostic certainty and algorithmic frameworks (Graus et al., 2016, 2021), which, while effective for many disorders, remain limited in conditions such as autoimmune psychosis (Pollak et al., 2020) or neuropsychiatric lupus (Emerson et al., 2023). Psychiatry, by contrast, is anchored in symptom-based nosology, yielding broad categories that fail to identify specific endophenotypes or neurobiologically distinct subtypes (Stephan et al., 2016). This disjunction is reinforced by high rates of comorbidity, without adequate models to determine whether these reflect shared mechanisms, causal interactions, or diagnostic overlap (Hesdorffer, 2016). Consequently, neuropsychiatry often lacks a conceptual bridge between symptom-based classification and neural models of dysfunction (Taslim et al., 2024).

The theoretical disjunction between disciplines is thus echoed in clinical practice, giving rise to two parallel and only partially integrated models of care: managing psychiatric symptoms in neurological patients, which dominates current services; and addressing neurological contributions to psychiatric illness, which is less common and under-resourced (Lykouras and Douzenis, 2008; Tian et al., 2023). These practices remain siloed within health systems, facing heterogeneous development and structural barriers across countries (Agrawal et al., 2015; Bhattacharya et al., 2015), particularly at the outpatient level (Agrawal et al., 2015).

The absence of approaches linking neurobiological variables with contextual and psychosocial factors undermines the translational potential of clinical neuroscience (Satel and Lilienfeld, 2014). Studies in this area disproportionately originate from high-income countries (HICs), limiting their global applicability. Psychosocial influences on aging and disease processes are well documented (Santamaría-García et al., 2021), yet remain underrepresented in neuropsychiatric models. “One-size-fits-all” frameworks fail to account for local variability, particularly in the Global South (Greene et al., 2022; Baez et al., 2023). In addition, subjective processes, such as meaning-making and identity, remain insufficiently incorporated into diagnostic and treatment paradigms (Ibáñez et al., 2023).

Neuropsychiatry still lacks a comprehensive integrative orientation. Although neurobiopsychosocial models are frequently invoked (Bolton, 2013), they rarely translate into multilevel, complexity-informed clinical tools. Methodological constraints, small sample sizes, and insufficient analytic depth limit current integrative approaches (Ibanez and Zimmer, 2023; Wu et al., 2023).

Finally, fragmentation in training exacerbates previously mentioned issues. Most neuropsychiatrists today are either self-taught (Sachdev P. S., 2005) or trained via research, with few structured clinical programs available worldwide (Bolton, 2013). Although core curricula have been proposed (Sachdev and Mohan, 2017), training is often confined to tertiary care settings, with minimal community-based integration (Agrawal et al., 2015), having repercussions in the process of construction of knowledge (Fulford et al., 2014).

### Toward a new neuropsychiatry: framework for an integrative paradigm

To address the longstanding conceptual and practical limitations of neuropsychiatry, diverse approaches across disciplines have begun to converge. Advances in neuroscience have facilitated closer integration between neurology and psychiatry, while insights from the humanities have reinforced the importance of subjective experience within neuroscientific perspectives (Fuchs, 2020; Kyzar and Denfield, 2023; Northoff and Ventura, 2025). These convergences support the need for a more coherent synthesis of clinical and research practices that can foster a dialogue between objective and subjective domains. In this section, we propose an epistemological structure aligned with contemporary research frameworks to lay the groundwork for a new neuropsychiatric paradigm, grounded in a non-reductive neurophilosophy informed by cooperative naturalism (Klar, 2021).

**Figure 2:**
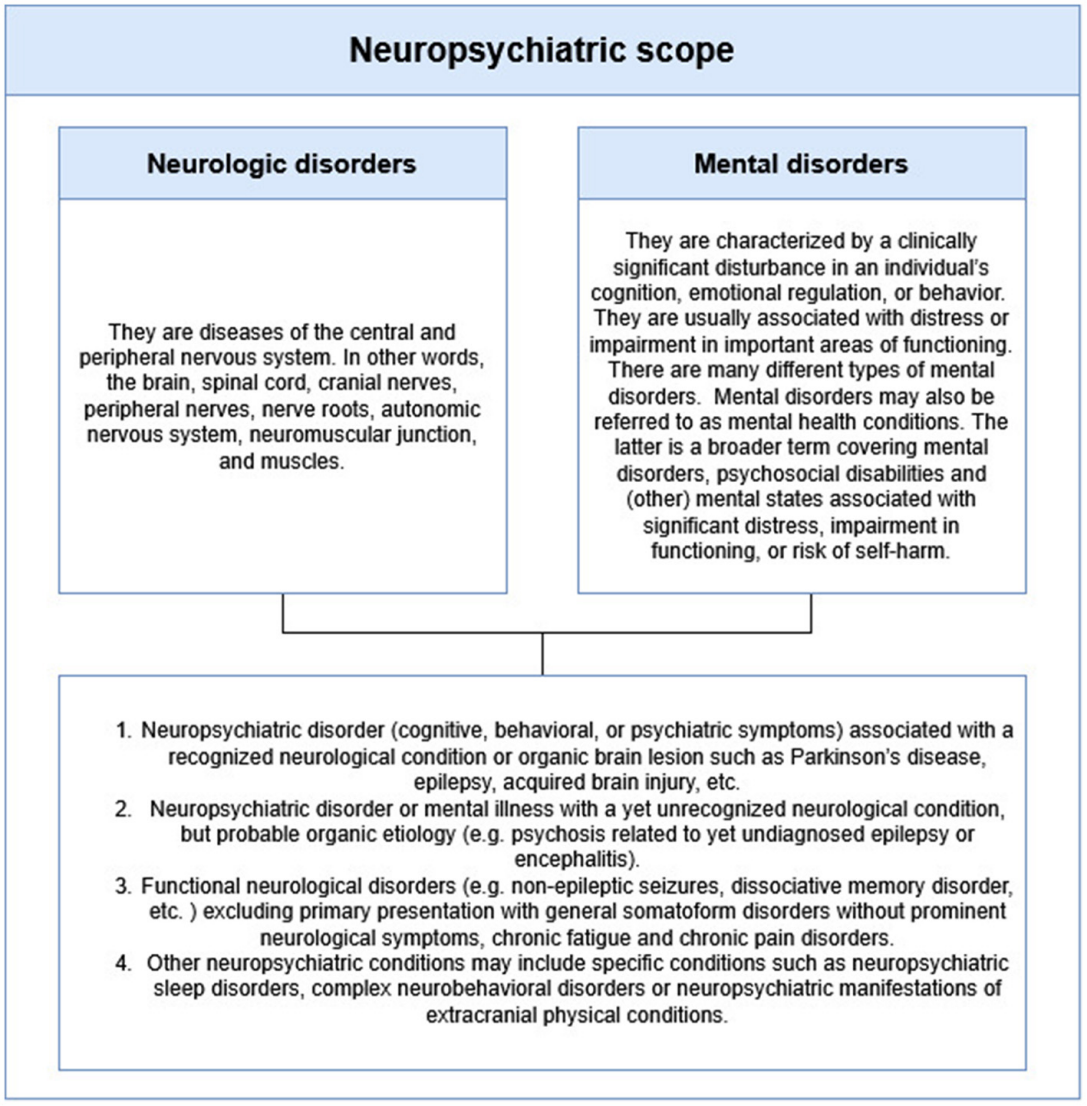
Neuropsychiatric scope. Neuropsychiatry requires epistemological boundaries to avoid being a “no man's land.” This figure contrasts WHO definitions of neurological and mental disorders with the scope proposed by Bhattacharya et al. (2015) highlighting the need for conceptual and clinical integration.

Neuropsychiatry is a hybrid discipline situated at the intersection of the humanities and natural sciences (Berríos, 2011), requiring a delimitation of its scope (Figure 2). It aims not only to explain brain states in neurological or psychiatric disorders but also aims to understand mental phenomena as dynamic, context-specific experiences imbued with emotional, volitional, and cognitive dimensions (Berríos, 2011). Unlike impairments in memory or language, mental symptoms acquire context-dependent meaning shaped by subjectivity and intersubjectivity (Fuchs, 2010). Thus, addressing mental phenomena within neurological disorders requires an inter-field approach; one that aims to integrate biological, environmental, and developmental dimensions while enabling a dialogue subjective experience and objective data (Kyzar and Denfield, 2023). This requires a domain and methodological pluralism (Klar, 2021).

Constructing knowledge within this paradigm requires viewing mental and body–brain domains as reciprocally interactive systems. A constructivist stance operationalizes this view through concept–fact iterativity, linking logical and empirical plausibility (Northoff, 2022). Neuropsychiatric inquiry must therefore incorporate empirical data while also integrating three levels of analysis: behavior, neuropsychological functions, and subjective experience (Ramírez-Bermúdez et al., 2024), highlighting the embodied nature of the human mind (Jingzhu and Qiaohua, 2018).

**Figure 3:**
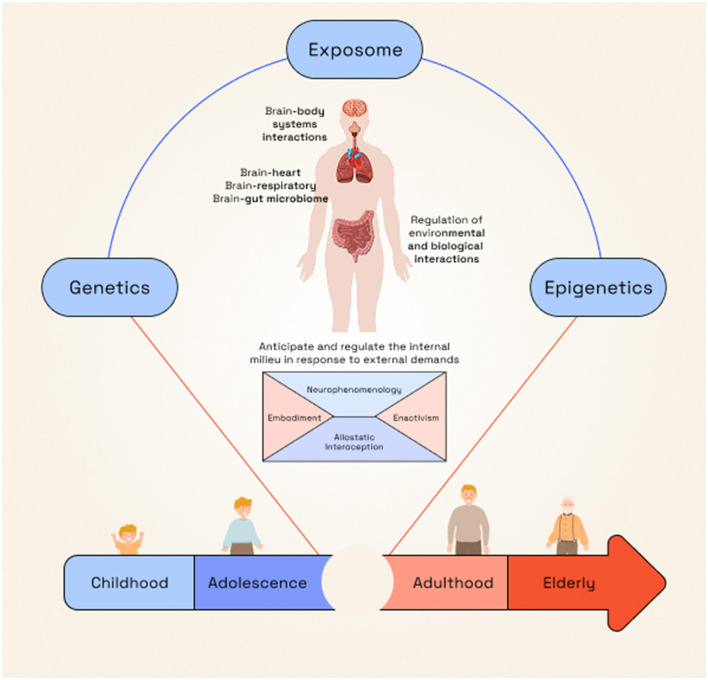
Framework for integrating subjective and objective perspectives. Neuropsychiatry requires regarding the person simultaneously as a subject and an object. Clinical and research assessments must therefore rely on frameworks that bridge subjective and objective perspectives, conceiving neurological and mental phenomena as components of an integrated body–brain system. Within this view, allostatic interoception offers a fundamental mechanism for understanding how the brain predicts and regulates bodily physiology while also giving rise to subjective experience, as proposed in constructed emotion theory (Barrett, 2017; Katsumi et al., 2022). Through this integrative process, allostasis shapes neurocognitive, affective, and social functioning (Santamaría-García et al., 2024). Allostatic interoception can be productively combined with research methodologies grounded in predictive coding and Bayesian brain models, with computational approaches (Castro Martínez and Santamaría-García, 2023; Santamaría-García et al., 2024). These frameworks enable the characterization of low-dimensional body states that typically lie outside awareness and that, upon becoming conscious, manifest as subjective experience (Aragona and Marková, 2015; Katsumi et al., 2022; Berrios and Marková, 2024). Accordingly, subjective accounts are indispensable for capturing the full complexity of neuropsychiatric phenomena. Neurophenomenology complements these models by characterizing the generic structures of experiences accessible to conscious awareness (Varela, 1996; Berkovich-Ohana et al., 2020), thus enabling mutual enrichment through the joint analysis of first-person and third-person data (Daly et al., 2024). To establish a clear dialogue between these levels: embodiment, as a way of subjective experience modulating biological variables, and enactivism, as a framework for conceptualizing person-environment interactions, provide crucial integrative bridges or points of convergence. Achieving this integration requires a systematic matching process between empirical and philosophical domains. Mental symptoms should be understood as empirical–ontological relations among brain, body, and world. In this context, philosophical concepts function as inputs that must be translated and operationalized for empirical investigation, thereby granting empirical plausibility to the conceptual framework and allowing for reciprocal refinement of its ontological claims. This bidirectional dialogue is essential for advancing a coherent, scientifically grounded neuropsychiatric paradigm.

This integration necessitates combining qualitative and quantitative approaches, bridging nomothetic and idiographic forms of knowledge (Slade, 2012). The tension between standardized measures and individual meaning can be productively addressed through constructionism, which acknowledges knowledge as negotiated and shaped by researchers and clinicians as active participants (Slade, 2012). This highlights the need for reflexive scientific practice within neuropsychiatry (Kamenova, 2010). Consequently, a non-reductive methodology is essential for developing ecological models integrating cognitive, affective, and social neuroscience with descriptive psychopathology (Stanghellini and Broome, 2014), neuropsychology, and phenomenology (Van Oudenhove and Cuypers, 2014). We put forward a proposal described in Figure 3.

Contemporary research frameworks exemplify this shift toward more integrative perspectives, due to the limitations of descriptive diagnostic categories that do not align with neuroscientific data, thus not capturing underlying mechanisms of dysfunction (Insel et al., 2010; Insel, 2014). This has produced a validity problem (Kendell and Jablensky, 2003). RDoC reconceptualizes mental disorders as brain disorders, promoting dimensional, translational models to identify dysfunctions in different units of analysis (Morris and Cuthbert, 2012). From an epistemological standpoint RDoC opens place to various research paradigms, enhancing a constructionist perspective (Fulford et al., 2014). Although promising, RDoC remains limited in its integration of social determinants and its applicability to neurological disorders.

HiTOP offers a dimensional approach to psychopathology that models symptom co-occurrence and provides a more precise phenotypic basis for neuroscientific research (Latzman et al., 2020). Nonetheless, HiTOP focuses on the content of subjective experience rather than its form, thus underrepresenting subjectivity (Stanghellini and Broome, 2014). Nonetheless, gaps remain in representing spectral conditions and sensorimotor domains fundamental to neuropsychiatry (Michelini et al., 2021). Together, RDoC and HiTOP offer promising, though incomplete, attempts to align biological and experiential perspectives.

Integrating dimensional frameworks can pave the way for neuropsychiatric phenotypes that capture multiple causal layers. These phenotypes may enhance neurobiological research and support individualized clinical decisions. Nonetheless, challenges remain in developing nosographic classifications that adequately reflect mechanistic diversity.

Early efforts have incorporated subjective measures (Kyzar and Denfield, 2023), aligning with the field's goal of reaching individualized, ecologically valid explanations. As research increasingly incorporates multilayered analyses, neuropsychiatrists are called to account for the multifactorial and developmental nature of brain–mind disorders (Bolton, 2013).

**Figure 4:**
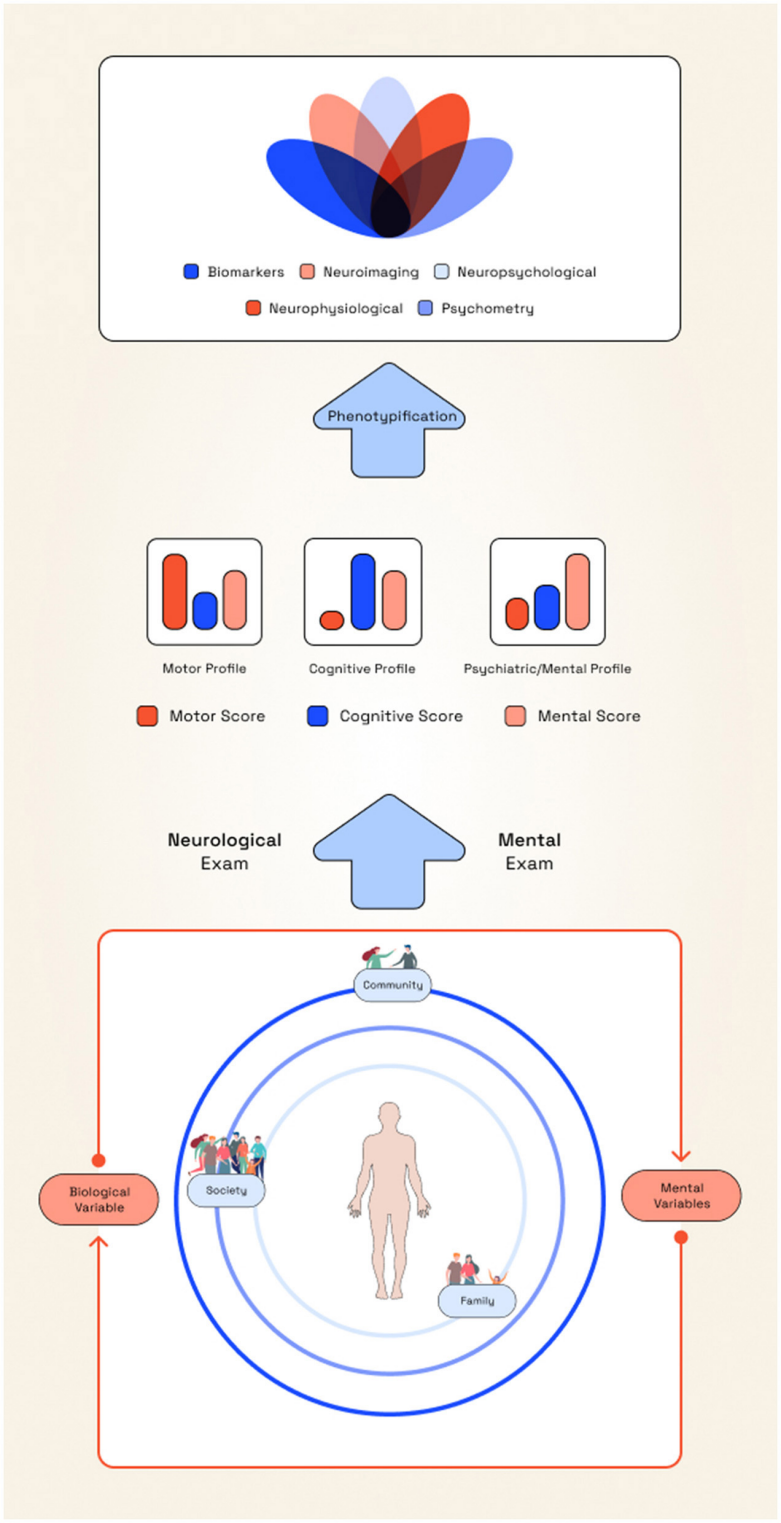
Proposed neuropsychiatric assessment model. The assessment and management of neuropsychiatric disorders should begin at the epistemological level, recognizing objective and subjective domains as inherently interrelated. When approaching a patient with a neuropsychiatric condition such as epilepsy, clinicians and researchers should adopt a constructivist stance, drawing on multiple theories to construct knowledge that is contextually meaningful and aligned with specific clinical or research aims. In doing so, neuropsychiatrists may employ theoretical models and clinical proposals that account for the heterogeneity of mental symptoms in neurological conditions, such as neurobehavioral profiles (Hermann et al., 2021). These profiles must be characterized through comprehensive clinical examinations, including both neurological and mental status assessments. Data acquisition emerges through the interaction between the clinician's or researcher's epistemological position, and the patient's lived experience (extended, enactive, embodied and embedded), thereby requiring ecological and context-sensitive evaluations. This approach allows distinct profiles to emerge, which can then guide efforts toward phenotypification. Deep phenotyping may be conducted across multiple levels, such as ion channel dysfunction, predictive brain processes, or subjective accounts, depending on the pragmatic needs of the assessment. The methods selected should facilitate inquiry at the relevant level of analysis, but also should have a dialogical construction to reach a conceptual ceiling. These different accounts must then be integrated, making use of their strengths while acknowledging their limitations. Each level offers distinct affordances for developing neurobiopsychosocial formulations. By adopting this approach, clinicians and researchers can select appropriate tools from their methodological repertoire and combine them with dimensional frameworks. This process of knowledge construction supports the development of individualized treatment plans and the design of research interventions, whether at the level of the individual or within the broader exposome, incorporating rehabilitative and psychosocial–community perspectives. Through this integrative framework, mental symptoms are understood as empirical–ontological relations among brain, body, and world.

To get closer to this vision, neuropsychiatry could adopt deep phenotyping strategies both at neurobiological and subjective levels. This involves the fine-grained characterization of individuals through multi-level integration of biological (genomic, epigenomic, proteomic, metabolic, neurological), environmental (exposomic), and subjective dimensions (Figure 4). These complex datasets enable the identification of meaningful subtypes and promote precision medicine approaches. Additionally, deep phenotyping could facilitate iterative conceptual analysis that recognizes the historical and context-sensitive construction of meaning, as exemplified by the functional–organic distinction (Bell et al., 2020; Chesterfield et al., 2023).

Notably, multiomics and connectomic models are now able to characterize how brain connectivity and systemic physiological processes (e.g., inflammation, autonomic regulation, metabolic activity) interact with structural social risk factors, trauma, and environmental exposures (Chen et al., 2019). Yet integrating these biological insights with subjective experience remains a major challenge. Mixed methods approaches are therefore necessary to achieve translational validity (Fulford et al., 2014).

Critically, this integrative proposal incorporates the subjective dimension as the lived, affectively charged experience of symptoms. The development of subjective patterns associated with deep phenotyping, *subjective phenotypes*, may allow neuropsychiatry to incorporate personal narratives and first-person data into multilevel analysis. This holistic integration promotes a nuanced science of the brain–body–mind–context interface and supports ethical, individualized, and clinically actionable care (Ritunnano, 2022), but also putting forward idiographic knowledge (Slade, 2012).

In this context, predictive coding and allostasis offer conceptual tools with implications at both biological and experiential levels (Santamaría-García et al., 2024). Predictive coding emphasizes the brain's capacity to generate and update models of the body and environment, while allostatic models explain how chronic dysregulation in stress responses may underpin both psychiatric and neurological symptoms, as sensed through interoception, making itself aware as an embodied affect, requiring categorization for the construction of an emotion (Seth and Friston, 2016; Barrett, 2017). These perspectives are especially useful in conditions like functional neurological disorders, where symptoms reflect dysregulated perception, prediction, and bodily control (Jungilligens et al., 2022). They also align with the brain's cytoarchitecture, offering a bridge between physical and psychological processes (Barrett, 2017; Katsumi et al., 2022).

In sum, a new neuropsychiatric orientation must transcend dualism and embrace complexity. Neuropsychiatry can offer a structured way to interpret disorders at the intersection of brain function, mental experience, and lived context through a constructionist epistemology that employs dimensional taxonomies, computational models, embodied cognitive science, and subjective data.

### Mobilizing innovation and integration through contextualized thinking

Overcoming the challenges of neuropsychiatry requires a systemic approach grounded in robust epistemology and integrative clinical-research translation (Berríos, 2007, 2011; Berrios, 2018). This effort must operate across academic, clinical, and regulatory levels and foster interdisciplinary models that combine subjective and objective perspectives (Berríos, 2007).

**Academic Formation and Clinical Training:** Training should be based in part to the UCNS core curriculum (Sachdev and Mohan, 2017), preparing clinicians to assess and treat neuropsychiatric symptoms, apply disease-modifying therapies, and work within multidisciplinary teams. Additionally, the syllabus components should be adapted to priorities within the region they are embedded (Kerr et al., 2025). Clinics should be collaborative, rooted in both psychiatry and neurology, and supported by biomarkers, neuroimaging, and precision medicine frameworks (Bateman et al., 2024). Tertiary care centers must align with primary and secondary levels and integrate with community mental health systems, as previous studies have highlighted as priority areas (Mary et al., 2017). Training should include applied neuroscience, computational methods (Castro Martínez and Santamaría-García, 2023), and bedside assessments, linking psychopathology with neurodiagnostics (Peña-Casanova et al., 2024), as a tool that enables a way to connect first-person subjective experience with brain functioning (Stanghellini and Broome, 2014). Thus, collaborative enterprises with other mental health professionals is a necessity.

This integrative education should also emphasize dynamic-functional anatomy (Northoff, 2008), neuromodulation (Siddiqi et al., 2021), and interdisciplinary rehabilitation, including leadership and task-sharing strategies (Le et al., 2022). Core questions remain on balancing neurology, psychiatry, and neuropsychiatric training in systems with limited resources.

**Clinical Practice:** Clinical expertise must integrate subjective experience with objective data, combining clinical skill with qualitative and quantitative research knowledge, grounded in an epistemically oriented mindset. Neuropsychiatrists should assess motor-cognitive-behavioral profiles of neuropsychiatric disorders and integrate neuroimaging and physiological data via translational models (Tozzi et al., 2024). This includes conditions such as gambling disorder (Szerman et al., 2020), functional neurological disorder (Jungilligens et al., 2022), and others with social and neurobiological risk factors (Koob and Volkow, 2016). A neurodevelopmental lens can aid in early detection and effective, low-cost interventions (Uhlhaas et al., 2023), supported by genetic, epigenetic, and developmental neuroscience (Nees et al., 2021).

Neuropsychology offers cognitive assessments aligned with functional neuroanatomy (Peña-Casanova et al., 2024) aligning with the needs of other clinical disciplines like neurosurgery, neurology and psychiatry, which are central to clinical evaluation through the use of theoretical models from normal cognitive psychology in a principled and testable manner (Halligan and David, 2001). In parallel, greater convergence across disciplines is needed around known gaps; specifically, specialized training and evidence-based assessment practices embedded within advocacy, to enhance integration into the broader medical landscape (Sweet et al., 2021). Neurosurgical collaboration, especially in refractory or surgically treated cases like epilepsy, is vital for identifying relevant neurocircuitry and managing postsurgical sequelae (Bauerle et al., 2023). Interdisciplinary practices can innovate therapeutic models (Pedrotty et al., 2021; Özge et al., 2023).

**Psychotherapy Enriched by Neuroscience:** Psychotherapy in neuropsychiatry must integrate subjective and neurobiological insights. The therapeutic alliance can be explored through affective, cognitive, and social neuroscience (Cammisuli and Castelnuovo, 2023), especially from an intersubjective perspective (Schilbach, 2016). Neuropsychoanalysis exemplifies how psychodynamic constructs can modulate and be modulated by brain function (Solms, 2013; Flores Mosri, 2021). The therapeutic potential of psychedelics further demands neurobiological frameworks to guide their integration (Reiff et al., 2020). However, despite these conceptual advances, clear and operational models specifying how such neuroscientific insights can be systematically translated into everyday psychotherapeutic practice remain largely underdeveloped.

**Neuromodulation:** Neuromodulation (rTMS, tDCS, DBS, VNS) is safe, and circuit-based (Hyde et al., 2022), integrating connectomic perspectives and advancing from circuit identification to system-level modeling (Tu et al., 2021). Understanding neuroanatomical mechanisms (Leaver et al., 2022) aids in localizing stimulation (Cocchi and Zalesky, 2018) and predicting outcomes (Pinna et al., 2018). Combining neuromodulation with psychotherapy and pharmacology represents a frontier in individualized treatment (Pedrotty et al., 2021).

**Research:** Neuropsychiatric research integrates first-, second-, and third-person perspectives, bridging psychiatry and neurology through computational tools (Castro Martínez and Santamaría-García, 2023), consortia (Thompson et al., 2020), and frameworks like RDoC and HiTOP (Michelini et al., 2021). Neurophenomenology (Berkovich-Ohana et al., 2020) links subjective experience with neuroscience, enabling personalized care models. These efforts can identify reversible causes or refine diagnoses. Longitudinal and idiographic methods enrich developmental trajectories (Piccirillo and Rodebaugh, 2019), while inclusion of diverse populations supports culturally grounded practices (Kleinman and Benson, 2006). Knowledge production becomes a collective, contextual process. Through this lens, social determinants gain relevance in neuroscience, prompting integration with humanities and social sciences.

## Discussion

This review offers a comprehensive and integrative framework for reconceptualizing neuropsychiatry as a distinct scientific field, grounded in naturalized philosophical currents, while maintaining a strong empirical foundation. Rather than a simple synthesis of neurology and psychiatry, we argue that neuropsychiatry is defined by its hybrid epistemology, which requires a constructivist posture and a multidimensional, interdisciplinary understanding of the dynamic interface between brain function and mental phenomena (Berrios and Marková, 2002; Berríos, 2011). From this standpoint, neuropsychiatry operates as a “third space”, conceptually and methodologically, where scientific models, subjective experience and contextual determinants converge in a dialogical and iterative manner.

Historically, the field has been shaped by heterogeneous and contingent processes. Enduring effects of Cartesian substance dualism (Berrios and Marková, 2002; Berrios, 2018; Thibaut, 2018) entrenched divisions between neurology and psychiatry, between objective and subjective accounts, and between reductionist vs non reductionist models of mental symptoms. Many of these challenges stem from epistemological limitations inherited from psychiatry, yet neuropsychiatry faces additional conceptual tensions arising from the particular niche of mental symptoms in neurological diseases. Moving beyond these binary frameworks requires a shift toward a constructivist and contextualized paradigm that incorporates developmental, dimensional, and socially informed models. This repositioning allows neuropsychiatry to leverage advances in neuroscience, including predictive coding and allostatic inference (Tu et al., 2021; Santamaría-García et al., 2024), alongside philosophical contributions from non reductive neurophilosophy (Van Oudenhove and Cuypers, 2014), neurophenomenology (Berkovich-Ohana et al., 2020), and enactive and embodied approaches (Krueger, 2021).

Consistent with this view, our analysis underscores that neuropsychiatric disorders frequently occupy a conceptual “no man's land” within traditional nosology systems. Strict categorical boundaries fail to capture the complexity of syndromic presentations. Dimensional and translational models (e.g., RDoC, HiTOP) (Latzman et al., 2020) provide more productive avenues for research and clinical practice, particularly when integrated with subjective and ecological data. These frameworks facilitate the delineation of neuropsychiatric phenotypes grounded in both brain-body dysfunction and lived experience, thereby enhancing diagnosis, rehabilitation, and treatment strategies (Estingoy, 2019), and conceptual analysis.

A central contribution of this review is its emphasis on contextualization. Cultural, technological, and socioeconomic factors shape how neuropsychiatric disorders are conceptualized, assessed, and treated (Berrios and Marková, 2002; Drouin et al., 2022). Thus, reflexivity becomes fundamental for framing those concepts. This is particularly relevant in regions like Latin America, where research capacity, training opportunities, and access to specialized care remain uneven (Baez et al., 2023; Ramírez Bermúdez et al., 2026). A globally sensitive neuropsychiatry must therefore integrate clinical neuroscience with local knowledge systems, low-cost diagnostic tools, and community-based practices. Such alignment improves ecological validity and helps mitigate persistent health inequities.

Training and clinical practice must reflect this complexity. Such programs should be vertically and horizontally integrated across levels of care (Mary et al., 2017), combining clinical exposure, neuroscientific knowledge, and cultural competence (Kleinman and Benson, 2006), with clinical competency with psychiatric and neurologic assessments in multidisciplinary teams and shared research infrastructures (Chemali, 2005). These systems should facilitate precision medicine approaches that account for genetic, epigenetic, and connectomic data, as well as social determinants and environmental exposures (Castro Martínez and Santamaría-García, 2023).

Our manuscript additionally advances the need for deep phenotyping frameworks capable of integrating body–brain biology, multi-omics, developmental trajectories, and environmental exposure. Neuropsychiatry is uniquely positioned to model the complexity of mental symptoms using computational approaches grounded in predictive processing and allostatic inference, while simultaneously engaging constructivist and relational perspectives (Barrett, 2017; Jungilligens et al., 2022). This integration enables more precise prediction, individualized interventions, and the development of system-level models that reflect the multilevel nature of neuropsychiatric disorders. It also contributes to the development of a “subjective phenotype”, a layer of lived experience that complements and enriches neurobiological and behavioral dimensions (Berkovich-Ohana et al., 2020), and fosters new methods for constructing knowledge (Figure 3).

This integrative and convergent view is exemplified by emerging dialogues between psychotherapy and neuroscience. Therapeutic encounters are inherently relational fields shaped by intersubjectivity, affectivity, and second-person neuroscience (Schilbach, 2016). Consequently, psychotherapeutic models should adopt a transdisciplinary orientation that incorporates these intersubjective processes while remaining grounded in, or explicitly informed by, neurobiological mechanisms. This is evident in novel modalities such as psychedelic-assisted psychotherapy (Reiff et al., 2020), which require conceptual frameworks capable of integrating neurobiological and subjective dimensions of the individual.

## Conclusions

Neuropsychiatry is poised to become a truly transdisciplinary field that unites empirical rigor with subjective insight and social relevance. Future development depends not on consolidating a single overarching theory but on adopting a constructive orientation that enables navigation through the multicausal, non-linear, and layered nature of mental disorders. This endeavor requires bridging basic and clinical science, objectivity and intersubjectivity, and global and local contexts. Such bridges define both the central challenge and the promise of contemporary neuropsychiatry. Rather than resolving longstanding ontological debates, neuropsychiatry can offer epistemic tools and methodological principles for integrating diverse explanatory levels, biological, psychological, phenomenological, and sociocultural, into clinically meaningful accounts.

By adopting pluralistic, non-reductive frameworks informed by dimensional models, deep phenotyping, embodied cognition, and subjective data, neuropsychiatry can advance toward individualized, context-sensitive, and ecologically valid approaches to understanding and treating mental symptoms.

This comprehensive, philosophically grounded, and practice-oriented vision outlines a roadmap for a neuropsychiatry that is scientifically robust, clinically innovative, and ethically attuned to diverse populations.

## References

[B1] Agrawal N. Bhattacharya R. Rickards H. (2015). Provision of neuropsychiatry services: variability and unmet need. BJPsych. Bull. 39, 297–301. doi: 10.1192/pb.bp.114.047324 26755990 PMC4706222

[B2] AMEDD Center of History and Heritage (n.d.). History of Neuropsychiatry in World War I. Available online at: https://achh.army.mil/history/book-wwi-neuropsychiatry-section2chapter1 (Accessed July 12, 2025).

[B3] ANPA (1988). American Neuropsychiatric Association.

[B4] Aragona M. Marková I. S. (2015). L'herméneutique de symptômes mentaux selon l'École de cambridge. Revista Latinoamericana de Psicopatologia Fundamental 18, 599–618. doi: 10.1590/1415-4714.2015v18n4p599.2

[B5] Arciniegas D. B. Kaufer D. I. (2006). Core curriculum for training in behavioral neurology and neuropsychiatry the joint advisory committee on subspecialty certification of the American Neuropsychiatric Association and the society for behavioral and cognitive neurology. J. Neuropsychiatry Clin. Neurosci. 18:6. doi: 10.1176/jnp.18.1.6 16525065

[B6] Baez S. Alladi S. Ibanez A. (2023). Global South research is critical for understanding brain health, ageing and dementia. Clin. Transl. Med. 13:e1486. doi: 10.1002/ctm2.1486 37987144 PMC10660824

[B7] Barrett L. F. (2017). The theory of constructed emotion: an active inference account of interoception and categorization. Soc. Cogn. Affect. Neurosci. 12, 1–23. doi: 10.1093/scan/nsx060 27798257 PMC5390700

[B8] Bateman J. R. Josephy-Hernandez S. Apostolova L. G. Benjamin S. Barrett A. M. Boeve B. F. . (2024). Promoting growth in behavioral neurology: a path forward. Cogn. Behav. Neurol. 37, 49–56. doi: 10.1097/WNN.0000000000000368 38717325

[B9] Bauerle L. Palmer C. Salazar C. A. Larrew T. Kerns S. E. Short E. B. . (2023). Neurosurgery for psychiatric disorders: reviewing the past and charting the future. Neurosurg. Focus 54:E8. doi: 10.3171/2022.11.FOCUS22622 36724525

[B10] Bell V. Wilkinson S. Greco M. Hendrie C. Mills B. Deeley Q. (2020). What is the functional/organic distinction actually doing in psychiatry and neurology? Wellcome Open Res. 5:138. doi: 10.12688/wellcomeopenres.16022.1 32685699 PMC7338913

[B11] Benjamin S. (2024). Dual residency training in neurology and psychiatry: history and current practice. J. Neuropsychiatry Clin. Neurosci. 36, 11–21. doi: 10.1176/appi.neuropsych.21110271 37727060

[B12] Berkovich-Ohana A. Dor-Ziderman Y. Trautwein F. M. Schweitzer Y. Nave O. Fulder S. . (2020). The Hitchhiker's guide to neurophenomenology—the case of studying self boundaries with meditators. Front. Psychol. 11:1680. doi: 10.3389/fpsyg.2020.01680 32793056 PMC7385412

[B13] Berrios G. Marková I. S. (2018). The epistemology of psychiatry. Revista Estudos do Século XX 19, 59–70. doi: 10.14195/1647-8622_19_4

[B14] Berríos G. E. (2007). What is neuropsychiatry? Rev. Colomb. Psiquiatr. 36, 9–14.

[B15] Berríos G. E. (2011). Psychiatry and its objects. Rev. Psiquiatr. Salud. Ment. 4, 179–182. doi: 10.1016/j.rpsm.2011.09.001 23446262

[B16] Berrios G. E. (2018). Historical epistemology of the body-mind interaction in psychiatry. Dialogues Clin. Neurosci. 20:5. doi: 10.31887/DCNS.2018.20.1/gberrios 29946206 PMC6016044

[B17] Berrios G. E. Marková I. S. (2002). The concept of neuropsychiatry a historical overview. J. Psychosom. Res. 53, 629–638. doi: 10.1016/S0022-3999(02)00427-0 12169337

[B18] Berrios G. E. Marková I. S. (2024). “The epistemology of psychiatry and mental symptoms: the Cambridge view,” in Phenomenological Neuropsychiatry, eds. A. L. Mishara, M. Moskalewics, M. A. Schwartz, and A. Kranjec (Cham: Springer Nature). doi: 10.1007/978-3-031-38391-5_4

[B19] Bhattacharya R. Rickards H. Agrawal N. (2015). Commissioning neuropsychiatry services: barriers and lessons. BJPsych. Bull. 39, 291–296. doi: 10.1192/pb.bp.114.047290 26755989 PMC4706214

[B20] BNPA (1987). British Neuropsychiatry Association.

[B21] Bogousslavsky J. Moulin T. (2009). From alienism to the birth of modern psychiatry: a neurological story? Eur. Neurol. 62, 257–263. doi: 10.1159/000235594 19690418

[B22] Bolton D. (2013). Should mental disorders be regarded as brain disorders? 21st century mental health sciences and implications for research and training. World Psychiatry 12, 24–25. doi: 10.1002/wps.20004 23471790 PMC3619181

[B23] Cammisuli D. M. Castelnuovo G. (2023). Neuroscience-based psychotherapy: a position paper. Front. Psychol. 14:1101044. doi: 10.3389/fpsyg.2023.1101044 36860785 PMC9968886

[B24] Castro Martínez J. C. Santamaría-García H. (2023). Understanding mental health through computers: an introduction to computational psychiatry. Front. Psychiatry 14:1092471. doi: 10.3389/fpsyt.2023.1092471 36824671 PMC9941647

[B25] Chemali Z. N. (2005). The essentials of neuropsychiatry: teaching residents and fellows the interface between psychiatry and neurology. Harv. Rev. Psychiatry 13, 312–315. doi: 10.1080/10673220500326466 16251169

[B26] Chen J. Liu J. Calhoun V. D. (2019). Translational potential of neuroimaging genomic analyses to diagnosis and treatment in mental disorders. Proc. IEEE 107, 912–927. doi: 10.1109/JPROC.2019.2913145 PMC7015534 32051642

[B27] Chesterfield A. Harvey J. Hendrie C. Wilkinson S. Juan N. V. S. Bell V. (2023). Meaning and role of functional-organic distinction: a study of clinicians in psychiatry and neurology services. Med. Humanit. 50, 170–178. doi: 10.1136/medhum-2023-012667 37968099

[B28] Churchland P. M. (1981). Eliminative materialism and the propositional attitudes. J. Philos. 78, 67–90. doi: 10.5840/jphil198178268

[B29] Cocchi L. Zalesky A. (2018). Personalized transcranial magnetic stimulation in psychiatry. Biol. Psychiatry Cogn. Neurosci. Neuroimaging 3, 731–741. doi: 10.1016/j.bpsc.2018.01.008 29571586

[B30] Coffey C. E. (1999). The American neuropsychiatric association: ten years of progress and a future of great promise. J. Neuropsychiatry Clin. Neurosci. 11, 8–18. doi: 10.1176/jnp.11.1.8 9990551

[B31] Crocq M.-A. Crocq L. (2000). From shell shock and war neurosis to posttraumatic stress disorder: a history of psychotraumatology. Dialogues Clin. Neurosci. 2, 47–55. doi: 10.31887/DCNS.2000.2.1/macrocq 22033462 PMC3181586

[B32] Daly A. Ritunnano R. Gallagher S. Kirmayer L. J. Van Dam N. Kleinman J. (2024). Examination of self patterns: framing an alternative phenomenological interview for use in mental health research and clinical practice. Front. Psychol. 15:1390885. doi: 10.3389/fpsyg.2024.1390885 39049941 PMC11267421

[B33] De Domenico M. Omodei E. Arenas A. (2016). Quantifying the diaspora of knowledge in the last century. Appl. Netw. Sci. 1. doi: 10.1007/s41109-016-0017-9 30533507 PMC6245217

[B34] Drouin E. Goetz C. G. Hautecoeur P. (2022). Neurology and psychiatry. complex historical relationships. Ann. Med. Psychol. (Paris) 180, 721–726. doi: 10.1016/j.amp.2022.07.021

[B35] Emerson J. S. Gruenewald S. M. Gomes L. Lin M. W. Swaminathan S. (2023). The conundrum of neuropsychiatric systemic lupus erythematosus: current and novel approaches to diagnosis. Front. Neurol. 14:1111769. doi: 10.3389/fneur.2023.1111769 37025200 PMC10070984

[B36] Estingoy P. (2019). The irresistible rise of an abolished discipline: neuropsychiatry in France (1968–2018)…. Ann. Med. Psychol. (Paris) 177, 459–463. doi: 10.1016/j.amp.2019.03.006

[B37] Ferrari A. J. Santomauro D. F. Mantilla A. M. Shadid J. Ashbaugh C. Erskine H. E. . (2022). Global, regional, and national burden of 12 mental disorders in 204 countries and territories, 1990–2019: a systematic analysis for the Global Burden of Disease Study 2019. Lancet Psychiatry 9, 137–150. doi: 10.1016/S2215-0366(21)00395-3 35026139 PMC8776563

[B38] Finucane G. Mohan A. Sachdev P. S. (2020). “Neuropsychiatric services in Australia and New Zealand,” in Oxford Textbook of Neuropsychiatry, eds. N. Agrawal, R. Faruqui, and M. Bodani (Oxford: Oxford University Press). doi: 10.1093/med/9780198757139.003.0045

[B39] Fischer B. A. (2012). A review of american psychiatry through its diagnoses: the history and development of the diagnostic and statistical manual of mental disorders. J. Nerv. Ment. Dis. 200, 1022–1030. doi: 10.1097/NMD.0b013e318275cf19 23197117

[B40] Flores Mosri D. (2021). Clinical applications of neuropsychoanalysis: hypotheses toward an integrative model. Front. Psychol. 12:718372. doi: 10.3389/fpsyg.2021.718372 34566799 PMC8458959

[B41] Friston K. FitzGerald T. Rigoli F. Schwartenbeck P. Pezzulo G. (2017). Active inference: a process theory. Neural. Comput. 29, 1–49. doi: 10.1162/NECO_a_00912 27870614

[B42] Fuchs T. (2010). Subjectivity and intersubjectivity in psychiatric diagnosis. Psychopathology 43, 268–274. doi: 10.1159/000315126 20516753

[B43] Fuchs T. (2020). The circularity of the embodied mind. Front. Psychol. 11:1707. doi: 10.3389/fpsyg.2020.01707 32903365 PMC7434866

[B44] Fulford K. W. M. Bortolotti L. Broome M. (2014). Taking the long view: an emerging framework for translational psychiatric science. World Psychiatry 13, 110–117. doi: 10.1002/wps.20139 24890054 PMC4102274

[B45] Goetz C. G. Chmura T. A. Lanska D. (2003). Part 1: The history of 19th century neurology and the American Neurological Association. Ann. Neurol. 53:S2. doi: 10.1002/ana.8888 12722087

[B46] Graus F. Titulaer M. J. Balu R. Benseler S. Bien C. G. Cellucci T. . (2016). A clinical approach to diagnosis of autoimmune encephalitis. Lancet Neurol. 15, 391–404. doi: 10.1016/S1474-4422(15)00401-9 26906964 PMC5066574

[B47] Graus F. Vogrig A. Muñiz-Castrillo S. Antoine J. C. G. Desestret V. Dubey D. . (2021). Updated diagnostic criteria for paraneoplastic neurologic syndromes. Neurol. (R) Neuroimmunol. Neuroinflamm. 8:e1014. doi: 10.1212/NXI.0000000000001014 34006622 PMC8237398

[B48] Greene A. S. Shen X. Noble S. Horien C. Hahn C. A. Arora J. . (2022). Brain–phenotype models fail for individuals who defy sample stereotypes. Nature 609:109. doi: 10.1038/s41586-022-05118-w 36002572 PMC9433326

[B49] Halligan P. W. David A. S. (2001). Cognitive neuropsychiatry: towards a scientific psychopathology. Nat. Rev. Neurosci. 2:209. doi: 10.1038/35058586 11256082

[B50] Heckers S. Engstrom E. J. Kendler K. S. (2022). “Manifestations of insanity”: Kraepelin's final views on psychiatric nosology in their historical context. Mol. Psychiatry 27, 328–334. doi: 10.1038/s41380-021-01232-9 34334789

[B51] Hermann B. P. Struck A. F. Busch R. M. Reyes A. Kaestner E. McDonald C. R. (2021). Neurobehavioural comorbidities of epilepsy: towards a network-based precision taxonomy. Nat. Rev. Neurol. 17:0123456789. doi: 10.1038/s41582-021-00555-z 34552218 PMC8900353

[B52] Hesdorffer D. C. (2016). Comorbidity between neurological illness and psychiatric disorders. CNS Spectr. 21, 230–238. doi: 10.1017/S1092852915000929 26898322

[B53] Hyde J. Carr H. Kelley N. Seneviratne R. Reed C. Parlatini V. . (2022). Efficacy of neurostimulation across mental disorders: systematic review and meta-analysis of 208 randomized controlled trials. Mol. Psychiatry 27, 2709–2719. doi: 10.1038/s41380-022-01524-8 35365806 PMC8973679

[B54] Ibáñez A. Kühne K. Miklashevsky A. Monaco E. Muraki E. Ranzini M. . (2023). Ecological meanings: a consensus paper on individual differences and contextual influences in embodied language. J. Cogn. 6. doi: 10.5334/joc.228 37841670 PMC10573819

[B55] Ibanez A. Zimmer E. R. (2023). Time to synergize mental health with brain health. Nat. Ment. Health 1, 441–443. doi: 10.1038/s44220-023-00086-0 38867916 PMC11168413

[B56] Insel T. Cuthbert B. Garvey M. Heinssen R. Pine D. S. Quinn K. . (2010). Research domain criteria (RDoC): toward a new classification framework for research on mental disorders. Am. J. Psychiatry 167. doi: 10.1176/appi.ajp.2010.09091379 20595427

[B57] Insel T. R. (2014). The nimh research domain criteria (rdoc) project: precision medicine for psychiatry. Am. J. Psychiatry 171, 395–397. doi: 10.1176/appi.ajp.2014.14020138 24687194

[B58] Jingzhu Z. Qiaohua R. (2018). Neurophenomenology: a perspective of scientific epistemology. Adv. Soc. Sci. Educ. Humanit. Res. 176, 392–396. doi: 10.2991/icmess-18.2018.87

[B59] Jungilligens J. Paredes-Echeverri S. Popkirov S. Barrett L. F. Perez D. L. (2022). A new science of emotion: implications for functional neurological disorder. Brain 145, 2648–2663. doi: 10.1093/brain/awac204 35653495 PMC9905015

[B60] Kamenova K. (2010). Why we should strive toward reflexive scientific practices in neuroscience. AJOB Neurosci. 1, 59–60. doi: 10.1080/21507740.2010.515963

[B61] Kandel E. R. (1998). A new intellectual framework for psychiatry. Am. J. Psychiatry 155, 457–469. doi: 10.1176/ajp.155.4.457 9545989

[B62] Katsumi Y. Theriault J. E. Quigley K. S. Barrett L. F. (2022). Allostasis as a core feature of hierarchical gradients in the human brain. Netw. Neurosci. 6, 1010–1031. doi: 10.1162/netn_a_00240 38800458 PMC11117115

[B63] Kendell R. Jablensky A. (2003). Distinguishing Between the Validity and Utility of Psychiatric Diagnoses. Available online at: http://ajp.psychiatryonline.org doi: 10.1176/appi.ajp.160.1.4 (Accessed July 12, 2025). 12505793

[B64] Kendler K. S. (2005). Toward a philosophical structure for psychiatry. Am. J. Psychiatry 162, 433–440. doi: 10.1176/appi.ajp.162.3.433 15741457

[B65] Kerr K. Burns L. Benjamin S. Joyce E. M. Singh J. Ramírez-Bermúdez J. . (2025). Unpacking neuropsychiatry and behavioural neurology training: scoping review of core syllabus components. BJPsych. Bull. doi: 10.1192/bjb.2025.10184. [Epub ahead of print]. 41312683 PMC13386283

[B66] Kim J. (1999). Making sense of emergence. Philos. Stud. 95, 3–36. doi: 10.1023/A:1004563122154

[B67] Klar P. (2021). What is neurophilosophy: do we need a non-reductive form? Synthese 199, 2701–2725. doi: 10.1007/s11229-020-02907-6

[B68] Kleinman A. Benson P. (2006). Anthropology in the clinic: the problem of cultural competency and how to fix it. PLoS Med 3, 1673–1676. doi: 10.1371/journal.pmed.0030294 17076546 PMC1621088

[B69] Koob G. F. Volkow N. D. (2016). Neurobiology of addiction: a neurocircuitry analysis. Lancet Psychiatry 3, 760–773. doi: 10.1016/S2215-0366(16)00104-8 27475769 PMC6135092

[B70] Krishnamoorthy E. S. Misra V. (2020). “Neuropsychiatry service provision in India and South Asia,” in Oxford Textbook of Neuropsychiatry, eds. N. Agrawal, R. Faruqui, and M. Bodani (Oxford: Oxford University Press). doi: 10.1093/med/9780198757139.003.0047

[B71] Krueger J. (2021). Enactivism, other minds, and mental disorders. Synthese 198, 365–389. doi: 10.1007/s11229-019-02133-9

[B72] Kyzar E. J. Denfield G. H. (2023). Taking subjectivity seriously: towards a unification of phenomenology, psychiatry, and neuroscience. Mol. Psychiatry 28, 10–16. doi: 10.1038/s41380-022-01891-2 36460728 PMC10130907

[B73] Latzman R. D. DeYoung C. G. The HiTOP Neurobiological Foundations Workgroup (2020). Using empirically-derived dimensional phenotypes to accelerate clinical neuroscience: the hierarchical taxonomy of psychopathology (HiTOP) framework. Neuropsychopharmacology 45, 1083–1085. doi: 10.1038/s41386-020-0639-6 32109934 PMC7235031

[B74] Le P. T. D. Eschliman E. L. Grivel M. M. Tang J. Cho Y. G. Yang X. . (2022). Barriers and facilitators to implementation of evidence-based task-sharing mental health interventions in low- and middle-income countries: a systematic review using implementation science frameworks. Implement. Sci. 17:4. doi: 10.1186/s13012-021-01179-z 35022081 PMC8756725

[B75] Leaver A. M. Espinoza R. Wade B. Narr K. L. (2022). Parsing the network mechanisms of electroconvulsive therapy. Biol. Psychiatry 92, 193–203. doi: 10.1016/j.biopsych.2021.11.016 35120710 PMC9196257

[B76] Levin J. (2023). “Functionalism,” in The Stanford Encyclopedia of Philosophy, eds. E. N. Zalta, and U. Nodelman (Stanford, CA: Stanford University).

[B77] Lykouras L. Douzenis A. (2008). Do psychiatric departments in general hospitals have an impact on the physical health of mental patients? Curr. Opin. Psychiatry 21, 398–402. doi: 10.1097/YCO.0b013e32830079d0 18520746

[B78] Martin J. B. (2002). The integration of neurology, psychiatry, and neuroscience in the 21st century. Am. J. Psychiatry 159, 695–704. doi: 10.1176/appi.ajp.159.5.695 11986119

[B79] Mary Q. Bhugra D. Tasman A. Pathare S. Priebe S. Smith S. . (2017). The WPA-lancet psychiatry commission on the future of psychiatry. Lancet Psychiatry 4, 775–818. doi: 10.1016/S2215-0366(17)30333-4 28946952

[B80] McGorry P. D. Hickie I. B. Kotov R. Schmaal L. Wood S. J. Allan S. M. . (2025). New diagnosis in psychiatry: beyond heuristics. Psychol. Med. 55:e26. doi: 10.1017/S003329172400223X 39911018 PMC12017357

[B81] Michelini G. Palumbo I. M. DeYoung C. G. Latzman R. D. Kotov R. (2021). Linking RDoC and HiTOP: a new interface for advancing psychiatric nosology and neuroscience. Clin. Psychol. Rev. 86:102025. doi: 10.1016/j.cpr.2021.102025 33798996 PMC8165014

[B82] Miyoshi K. (2020). “Neuropsychiatry services in Japan,” in Oxford Textbook of Neuropsychiatry, eds. N. Agrawal, R. Faruqui, and M. Bodani (Oxford: Oxford University Press). doi: 10.1093/med/9780198757139.003.0046

[B83] Molina-Ruiz R. Nakagami Y. Mörkl S. Vargas M. Shalbafan M. Chang J. P. -C. . (2024). Training in neuropsychiatry: views of early career psychiatrists from across the world. BJPsych. Bull. 48, 78–84. doi: 10.1192/bjb.2023.32 37395121 PMC10985715

[B84] Morris S. E. Cuthbert B. N. (2012). Research domain criteria: cognitive systems, neural circuits, and dimensions of behavior. Dialogues Clin. Neurosci. 14, 29–37. doi: 10.31887/DCNS.2012.14.1/smorris 22577302 PMC3341647

[B85] Nees F. Deserno L. Holz N. E. Romanos M. Banaschewski T. (2021). Prediction along a developmental perspective in psychiatry: how far might we go? Front. Syst. Neurosci. 15:e670404. doi: 10.3389/fnsys.2021.670404 34295227 PMC8290854

[B86] Nichols E. Szoeke C. E. I. Vollset S. E. Abbasi N. Abd-Allah F. Abdela J. . (2019). Global, regional, and national burden of Alzheimer's disease and other dementias, 1990–2016: a systematic analysis for the Global Burden of Disease Study 2016. Lancet Neurol. 18, 88–106. doi: 10.1016/S1474-4422(18)30403-4 30497964 PMC6291454

[B87] Northoff G. (2008). Neuropsychiatry: an old discipline in a new gestalt bridging biological psychiatry, neuropsychology, and cognitive neurology. Eur. Arch. Psychiatry Clin. Neurosci. 258, 226–238. doi: 10.1007/s00406-007-0783-6 18297424

[B88] Northoff G. (2014). “Brain and philosophy: neurophilosophy,” in Minding the Brain: A Guide to Philosophy and Neuroscience (London: Palgrave Macmillan). doi: 10.1007/978-1-137-40605-7_5

[B89] Northoff G. (2022). Non-reductive neurophilosophy-what is it and how it can contribute to philosophy. J. NeuroPhilosophy 1. doi: 10.5281/zenodo.6637657

[B90] Northoff G. Ventura B. (2025). Bridging the gap of brain and experience—converging neurophenomenology with spatiotemporal neuroscience. Neurosci. Biobehav. Rev. 173:106139. doi: 10.1016/j.neubiorev.2025.106139 40204159

[B91] Onnela J. P. Rauch S. L. (2016). Harnessing smartphone-based digital phenotyping to enhance behavioral and mental health. Neuropsychopharmacology 41, 1691–1696. doi: 10.1038/npp.2016.7 26818126 PMC4869063

[B92] Özge A. Domaç F. M. Tekin N. Sünbül E. A. Öksüz N. Atalar A. Ç. . (2023). One patient, three providers: a multidisciplinary approach to managing common neuropsychiatric cases. J. Clin. Med. 12:5754. doi: 10.3390/jcm12175754 37685821 PMC10488785

[B93] Park S. C. (2019). Karl Jaspers' general psychopathology (Allgemeine Psychopathologie) and its implication for the current psychiatry. Psychiatry Investig. 16, 99–108. doi: 10.30773/pi.2018.12.19.2 30808115 PMC6393754

[B94] Parnas J. Sass L. A. Zahavi D. (2013). Rediscovering psychopathology: the epistemology and phenomenology of the psychiatric object. Schizophr. Bull. 39, 270–277. doi: 10.1093/schbul/sbs153 23267191 PMC3576163

[B95] Pedrotty M. Wong T. S. Wilde E. A. Bigler E. D. Laatsch L. K. (2021). Application of neuropsychology and imaging to brain injury and use of the integrative cognitive rehabilitation psychotherapy model. NeuroRehabilitation 49, 307–327. doi: 10.3233/NRE-218028 34420990

[B96] Peña-Casanova J. Sánchez-Benavides G. Sigg-Alonso J. (2024). Updating functional brain units: insights far beyond Luria. Cortex. doi: 10.1016/j.cortex.2024.02.004 38492440

[B97] Piccirillo M. L. Rodebaugh T. L. (2019). Foundations of idiographic methods in psychology and applications for psychotherapy. Clin. Psychol. Rev. 71, 90–100. doi: 10.1016/j.cpr.2019.01.002 30665765 PMC11130566

[B98] Pies R. (2005). Why psychiatry and neurology cannot simply merge. J. Neuropsychiatry Clin. Neurosci. 17, 304–309. doi: 10.1176/jnp.17.3.304 16179651

[B99] Pinna M. Manchia M. Oppo R. Scano F. Pillai G. Loche A. P. . (2018). Clinical and biological predictors of response to electroconvulsive therapy (ECT): a review. Neurosci. Lett. 669, 32–42. doi: 10.1016/j.neulet.2016.10.047 27793702

[B100] Pollak T. A. Lennox B. R. Müller S. Benros M. E. Prüss H. Tebartz van Elst L. . (2020). Autoimmune psychosis: an international consensus on an approach to the diagnosis and management of psychosis of suspected autoimmune origin. Lancet Psychiatry 7, 93–108. doi: 10.1016/S2215-0366(19)30290-1 31669058

[B101] Poole N. A. Bolton D. (2020). “Philosophy and neuropsychiatry,” in Oxford Textbook of Neuropsychiatry, eds. N. Agrawal, R. Faruqui, and M. Bodani (Oxford: Oxford University Press). doi: 10.1093/med/9780198757139.003.0004

[B102] Ramírez Bermúdez J. Castro-Suarez S. D'Alessio L. Holguín Lew J. Makarem Oliveira L. Sanches Yassuda M. . (2026). Towards a Latin American neuropsychiatry: challenges and opportunities. Lancet Reg. Health Am. 54:101322. doi: 10.1016/j.lana.2025.101322 41438237 PMC12719751

[B103] Ramírez-Bermúdez J. Juarez F. P. G. Aliseda A. (2024). Neuropsychiatric constructs as bridges between psychopathology and neuropathology: a medical perspective. Rev. Philos. Psychol. doi: 10.1007/s13164-024-00759-4

[B104] Ramirez-Bermudez J. Perez-Esparza R. Aguilar-Venegas L. C. Sachdev P. (2017). Neuropsychiatry: towards a philosophy of praxis. Rev. Colomb. Psiquiatr. 46, 28–35. doi: 10.1016/j.rcp.2017.07.001 29037336

[B105] Reiff C. M. Richman E. Nemeroff C. B. Carpenter L. L. Widge A. S. Rodriguez C. I. . (2020). Psychedelics and psychedelic-assisted psychotherapy: clinical implications. Am. J. Psychiatry 177:391. doi: 10.1176/appi.ajp.2019.19010035 32098487

[B106] Ritunnano R. (2022). Overcoming hermeneutical injustice in mental health: a role for critical phenomenology. J. Br. Soc. Phenomenol. 53, 243–260. doi: 10.1080/00071773.2022.2031234

[B107] Sachdev P. Mohan A. (2017). An international curriculum for neuropsychiatry and behavioural neurology. Rev. Colomb. Psiquiatr. 46, 18–27. doi: 10.1016/j.rcp.2017.05.001 29037334

[B108] Sachdev P. S. (2005). Whither neuropsychiatry? J. Neuropsychiatry Clin. Neurosci. 17, 140–141. doi: 10.1176/appi.neuropsych.17.2.140 15939966

[B109] Sachdev P. S. (2005). Whither neuropsychiatry? J. Neuropsychiatry Clin. Neurosci. 17:140. doi: 10.1176/appi.neuropsych.17.2.140 15939966

[B110] Santamaría-García H. Baez S. Aponte-Canencio D. M. Pasciarello G. O. Donnelly-Kehoe P. A. Maggiotti G. . (2021). Uncovering social-contextual and individual mental health factors associated with violence via computational inference. Patterns 2:100176. doi: 10.1016/j.patter.2020.100176 33659906 PMC7892360

[B111] Santamaría-García H. Migeot J. Medel V. Hazelton J. L. Teckentrup V. Romero-Ortuno R. . (2024). Allostatic interoceptive overload across psychiatric and neurological conditions. Biol. Psychiatry 97:28. doi: 10.1016/j.biopsych.2024.06.024 38964530 PMC12012852

[B112] Satel S. Lilienfeld S. O. (2014). Addiction and the brain-disease fallacy. Front. Psychiatry 4:141. doi: 10.3389/fpsyt.2013.00141 24624096 PMC3939769

[B113] Schilbach L. (2016). Towards a second-person neuropsychiatry. Philos. Trans. R. Soc. B Biol. Sci. 371:20150081. doi: 10.1098/rstb.2015.0081 26644599 PMC4685526

[B114] Scull A. (2017). Book review: the hunting of the snark: a search for the history of neuropsychiatry. Brain 140, 1166–1169. doi: 10.1093/brain/awx032

[B115] Seth A. K. Friston K. J. (2016). Active interoceptive inference and the emotional brain. Philos. Trans. R. Soc. B Biol. Sci. 371:20160007. doi: 10.1098/rstb.2016.0007 28080966 PMC5062097

[B116] Siddiqi S. H. Schaper F. L. W. V. J. Horn A. Hsu J. Padmanabhan J. L. Brodtmann A. . (2021). Brain stimulation and brain lesions converge on common causal circuits in neuropsychiatric disease. Nat. Hum. Behav. 5, 1707–1716. doi: 10.1038/s41562-021-01161-1 34239076 PMC8688172

[B117] Slade M. (2012). “The epistemological basis of personal recovery,” in Recovery of People with Mental Illness. Philosophical and Related Perspective, ed. A. Rudnick (Oxford: Oxford University Press). doi: 10.1093/med/9780199691319.003.0006

[B118] Slud Brofman G. Brusco L. I. (2020). “Neuropsychiatric services in South America,” in Oxford Textbook of Neuropsychiatry, eds. N. Agrawal, R. Faruqui, and M. Bodani (Oxford: Oxford University Press). doi: 10.1093/med/9780198757139.003.0049

[B119] Solms M. (2013). The conscious Id. Neuropsychoanalysis 15, 5–19. doi: 10.1080/15294145.2013.10773711

[B120] Stanghellini G. Broome M. R. (2014). Psychopathology as the basic science of psychiatry. Br. J. Psychiatry 205, 169–170. doi: 10.1192/bjp.bp.113.138974 25179621

[B121] Steinmetz J. D. Seeher K. M. Schiess N. Nichols E. Cao B. Servili C. . (2024). Global, regional, and national burden of disorders affecting the nervous system, 1990–2021: a systematic analysis for the Global Burden of Disease Study 2021. Lancet Neurol. 23, 344–381. doi: 10.1016/S1474-4422(24)00038-3 38493795 PMC10949203

[B122] Stephan K. E. Bach D. R. Fletcher P. C. Flint J. Frank M. J. Friston K. J. . (2016). Charting the landscape of priority problems in psychiatry, part 1: classification and diagnosis. Lancet Psychiatry 3, 77–83. doi: 10.1016/S2215-0366(15)00361-2 26573970

[B123] Sweet J. J. Klipfel K. M. Nelson N. W. Moberg P. J. (2021). Professional practices, beliefs, and incomes of U.S. neuropsychologists: the AACN, NAN, SCN 2020 practice and “salary survey.” Clin. Neuropsychol. 35, 7–80. doi: 10.1080/13854046.2020.1849803 33375892

[B124] Szerman N. Ferre F. Basurte-Villamor I. Vega P. Mesias B. Marín-Navarrete R. . (2020). Gambling dual disorder: a dual disorder and clinical neuroscience perspective. Front. Psychiatry 11:589155. doi: 10.3389/fpsyt.2020.589155 33329137 PMC7732481

[B125] Taslim S. Shadmani S. Saleem A. R. Kumar A. Brahma F. Blank N. . (2024). Neuropsychiatric disorders: bridging the gap between neurology and psychiatry. Cureus. 16:e51655. doi: 10.7759/cureus.51655 38313968 PMC10838116

[B126] Thibaut F. (2018). The mind-body Cartesian dualism and psychiatry. Dialogues Clin. Neurosci. 20:3. doi: 10.31887/DCNS.2018.20.1/fthibaut 29946205 PMC6016047

[B127] Thompson P. M. Jahanshad N. Ching C. R. K. Salminen L. E. Thomopoulos S. I. Bright J. . (2020). ENIGMA and global neuroscience: a decade of large-scale studies of the brain in health and disease across more than 40 countries. Transl. Psychiatry 10:100. doi: 10.1038/s41398-020-0705-1 32198361 PMC7083923

[B128] Tian Y. E. Di Biase M. A. Mosley P. E. Lupton M. K. Xia Y. Fripp J. . (2023). Evaluation of brain-body health in individuals with common neuropsychiatric disorders. JAMA Psychiatry 80:567. doi: 10.1001/jamapsychiatry.2023.0791 37099313 PMC10134046

[B129] Tozzi L. Zhang X. Pines A. Olmsted A. M. Zhai E. S. Anene E. T. . (2024). Personalized brain circuit scores identify clinically distinct biotypes in depression and anxiety. Nat. Med. 30:2076. doi: 10.1038/s41591-024-03057-9 38886626 PMC11271415

[B130] Trimble M. (2016). The intentional brain—a short history of neuropsychiatry. CNS Spectr. 21, 223–229. doi: 10.1017/S1092852916000195 27322690

[B131] Tu P. -C. Chen M. -H. Chang W. -C. Kao Z. -K. Hsu J. -W. Lin W. -C. . (2021). Identification of common neural substrates with connectomic abnormalities in four major psychiatric disorders: a connectome-wide association study. Eur. Psychiatry 64:e8. doi: 10.1192/j.eurpsy.2020.106 33267917 PMC8057470

[B132] Tyler K. York G. K. Steinberg D. A. Okun M. S. Steinbach M. Satran R. . (2003). Part 2: history of 20th century neurology: decade by decade. Ann. Neurol. 53, S27–S45. doi: 10.1002/ana.1346 12722088

[B133] Uhlhaas P. J. Davey C. G. Mehta U. M. Shah J. Torous J. Allen N. B. . (2023). Towards a youth mental health paradigm: a perspective and roadmap. Mol. Psychiatry 28, 3171–3181. doi: 10.1038/s41380-023-02202-z 37580524 PMC10618105

[B134] Van Oudenhove L. Cuypers S. (2014). The relevance of the philosophical “mind-body problem” for the status of psychosomatic medicine: a conceptual analysis of the biopsychosocial model. Med. Health Care Philos. 17, 201–213. doi: 10.1007/s11019-013-9521-1 24443097

[B135] Van Oudenhove L. Cuypers S. E. (2010). The philosophical “mind-body problem” and its relevance for the relationship between psychiatry and the neurosciences. Perspect. Biol. Med. 53, 545–557. doi: 10.1353/pbm.2010.0012 21037408

[B136] Varela F. J. (1996). Neurophenomenology: a methodological remedy for the hard problem. J. Conscious. Stud. 3, 330–349.

[B137] Wilson M. (1993). DSM-III and the transformation of American Psychiatry: a history. Am. J. Psychiatry 150, 399–410. doi: 10.1176/ajp.150.3.399 8434655

[B138] Wu J. Li J. Eickhoff S. B. Scheinost D. Genon S. (2023). The challenges and prospects of brain-based prediction of behaviour. Nat. Hum. Behav. 7:1255. doi: 10.1038/s41562-023-01670-1 37524932

